# Smoking, alcohol consumption, physical activity, and family history and the risks of acute myocardial infarction and unstable angina pectoris: a prospective cohort study

**DOI:** 10.1186/1471-2261-11-13

**Published:** 2011-03-24

**Authors:** Audrey HH Merry, Jolanda MA Boer, Leo J Schouten, Edith JM Feskens, WM Monique Verschuren, Anton PM Gorgels, Piet A van den Brandt

**Affiliations:** 1 Department of Epidemiology, CAPHRI School for Public Health and Primary Care, Maastricht University, Maastricht, the Netherlands; 2 National Institute for Public Health and the Environment (RIVM), Bilthoven, the Netherlands; 3 Department of Epidemiology, GROW-School for Oncology and Developmental Biology, Maastricht University, Maastricht, the Netherlands; 4 Division of Human Nutrition, Wageningen University, Wageningen, the Netherlands; 5 Department of Cardiology, University Hospital Maastricht, Maastricht, the Netherlands

## Abstract

**Background:**

Few studies investigated the association between smoking, alcohol consumption, or physical activity and the risk of unstable angina pectoris (UAP), while the strength of these associations may differ compared to other coronary diseases such as acute myocardial infarction (AMI). Therefore, we investigated whether the associations of these lifestyle factors with UAP differed from those with AMI. Additionally, we investigated whether these effects differed between subjects with and without a family history of myocardial infarction (MI).

**Methods:**

The CAREMA study consists of 21,148 persons, aged 20-59 years at baseline and randomly sampled from the Maastricht region in 1987-1997. At baseline, all participants completed a self-administered questionnaire. After follow-up of maximally 16.9 years, 420 AMI and 274 UAP incident cases were registered. Incidence rate ratios (RRs) were estimated using Cox proportional hazards models.

**Results:**

For both diseases, smoking increased the risk while alcohol consumption was associated with a protective effect. Associations with both risk factors were stronger for AMI than UAP, although this difference was only statistically significant for smoking. In men, an inverse association was found with physical activity during leisure time which seemed to be stronger for the risk of UAP than of AMI. On the contrary, physical activity during leisure time was associated with an increased risk of both AMI and UAP in women which seemed to be weaker for UAP than for AMI. Except for occupational physical activity in women, no significant interactions on a multiplicative scale were found between the lifestyle factors and family history of MI. Nevertheless, the highest risks were found in subjects with both a positive family history and the most unfavorable level of the lifestyle factors.

**Conclusions:**

The strength of the associations with the lifestyle factors did not differ between AMI and UAP, except for smoking. Furthermore, the effects of the lifestyle factors on the risk of both coronary diseases were similar for subjects with and without a positive family history.

## Background

Lifestyle factors such as smoking, physical inactivity, and alcohol consumption play an important role in the etiology of coronary heart diseases (CHD) [1]. Although these factors are well-known risk factors for CHD, only few studies investigated the associations between the risk of (unstable) angina pectoris and smoking status [2,3], alcohol consumption [4-8], or physical inactivity [9-11]. In contrast, the influence of these lifestyle factors on the risk of total CHD or acute myocardial infarction (AMI) is thoroughly investigated e.g. [12-18]. Although patients with unstable angina pectoris (UAP) have better survival rates than patients with AMI, their cardiac rehospitalization rates and quality of life scores are similar or even worse [19]. In addition, cases with UAP are more often female and are older compared with AMI cases [20,21]. The clinical presentation with UAP or AMI, especially ST elevation myocardial infarctions (STEMI), depends on the degree of occlusion of the coronary artery. Thrombotic and fibrinolytic processes may play a role in this difference in pathophysiology. Studies have shown that lifestyle factors are associated with haemostatic disturbances affecting these processes [22-24], which suggests that the strength of the associations with etiological factors may be different for UAP and AMI, in particular STEMI.

In several studies, family history of premature CHD or myocardial infarction (MI) was found to be an independent risk factor for coronary diseases [25-27]. Because subjects with a positive family history may be more susceptible to other risk factors, the associations between lifestyle factors and the risk of coronary diseases may be different in subjects with and without a positive family history. However, only few studies investigated a possible synergistic effect between family history and other risk factors for CHD [27-30].

To elucidate further the associations between lifestyle factors and the risk of UAP and to see whether these associations indeed differ in strength between AMI and UAP, the objective of this study was to investigate the effects of smoking, alcohol consumption, and physical inactivity on the risks of AMI and UAP as separate endpoints. A second objective was to study the association between family history of MI and the risks of these coronary endpoints. In addition, we investigated whether the associations between the lifestyle factors and the risks of AMI and UAP differed for people with and without a family history of premature MI.

## Methods

### CAREMA study

The study participants, living in the Maastricht region, were derived from two large monitoring projects in the Netherlands: the Monitoring Project on Cardiovascular Risk Factors (PPHVZ) [31] and the Monitoring Project on Chronic Disease Risk Factors (MORGEN) [32]. Between 1987 and 1997, a new random sample of people aged 20-59 years was selected each year from the population registries of Maastricht and surrounding municipalities, i.e. Eijsden, Margraten, Meerssen, and Valkenburg aan de Geul. These samples were stratified according to sex and 5-year age groups to obtain equal numbers in each age category. From 1987 until 1997, 21,662 men and women were included in this study (response rate 43%). The monitoring studies were approved by the Medical Ethics Committee of Leiden University and the Dutch Organization for Applied Research (TNO).

### Baseline data

At baseline, all participants filled in a self-administered questionnaire on demographics, medical history, family history of MI, and lifestyle factors such as smoking, alcohol consumption, and physical activity. Furthermore, their blood pressure, height, and weight were measured during a medical examination by trained staff members who were instructed by the same physician. Non-fasting blood samples were taken for the determination of total and HDL cholesterol levels.

For smoking, data was collected about the subject's smoking status (never, ex, or current), frequency (number of cigarettes/day) and duration (number of years of smoking). For ex-smokers, time since quitting was calculated using information about the starting age and duration of smoking.

For alcohol consumption, subjects were asked to report their drinking frequency: never, ex, occasional (<1 glass/week), or regular (≥1 glass/week). In addition, regular drinkers were asked about the number of glasses of beer, wine including fortified wines, and liquor they consumed per week. Furthermore, subjects reported their amount of physical activity during work, if applicable, and during leisure time.

For family history of MI, participants reported whether their father or mother ever had an MI including the age at diagnosis. A premature MI was defined as a diagnosis ≤60 years for the father and ≤65 years for the mother [33]. Based on these data, three variables were created for family history of MI: number of parents affected, parental history of premature MI, and a combined variable based on the previous two variables.

### Follow-up

A migration and mortality follow-up was performed by record linkage of the cohort to the Municipal Population Registries. Of all participants, 21,148 (97.6%) had given written informed consent to retrieve information from Municipal Population Registries and their general practitioner or specialist. During follow-up until December 31^st ^2003, 2,106 participants (10.0%) had migrated out of the Maastricht region, 621 participants (2.9%) had emigrated abroad, 791 participants (3.7%) had died, and 12 participants (0.1%) were lost to follow-up.

Incident cases of AMI and UAP were identified in two ways, 1) by linkage to the Cardiologic Information System (CIS) of the University Hospital Maastricht (UHM), and 2) by linkage to the causes of death registry of Statistics Netherlands. Using the CIS, incident cases with AMI or UAP were based on the first recorded clinical diagnosis during follow-up. This clinical diagnosis was mostly mentioned in the report to the general practitioner and was validated using the additional information in CIS including ECG findings, enzyme levels, and catheterization results. Linkage to the CIS was approved by the Medical Ethical Committee of the UHM. From the causes of death registry of Statistics Netherlands, all incident cases were identified using the following International Classification of Diseases (ICD) codes: ICD-9 410 and ICD-10 I21-I22 for AMI; ICD-9 413 and ICD-10 I20 for UAP. Statistics Netherlands receives the death certificate including a certificate with the cause of death for all Dutch inhabitants who deceased. This certificate is filled in by the physician who declares a person's death. The encoding of these cause of death certificates by Statistics Netherlands occurs according to the guidelines of the World Health Organization using the ICD coding system. In case of indistinct or incomplete data, the concerning physician is contacted for more clarity. To guarantee the quality of these data, quality controls and corrections are made by Statistics Netherlands for all of the primary causes of death and, to a lesser extent, for the secondary causes of death.

End of follow-up was determined by a clinical diagnosis of AMI, UAP, a coronary bypass artery grafting, or a percutaneous coronary intervention, migration out of the Maastricht region, emigration, death, or censoring at December 31^st ^2003, whichever occurred first. Person-time at risk was calculated from baseline until end of follow-up. More details about the study and follow-up are described in a previous publication [34].

### Statistical analyses

Incidence rate ratios (RR) and 95% confidence intervals (CI) were estimated in Cox proportional hazards models using the Stata statistical software package 9.2 (Stata Corporation, College Station, TX, USA), after testing of the proportional hazards assumption using the scaled Schoenfeld residuals and by checking the log-log survival curves. For both AMI and UAP, this assumption was not violated by the main variables, except for time since quitting and the UAP risk. However, stratified analyses according to follow-up period were not possible because of the small number of UAP cases in the categories for time since quitting. Two-sided *p *values are reported throughout this paper. To obtain *p *values for dose-response trends, ordinal variables were fitted as continuous terms in the models.

All risk estimates were adjusted for age at baseline (years), sex, baseline cohort (PPHVZ or MORGEN), smoking status, frequency, and duration (except for the models on smoking), and total alcohol consumption in glasses/day (except for the models on alcohol). Furthermore, the models for smoking, alcohol consumption, and physical activity were additionally adjusted for educational level (primary school/junior vocational education, secondary vocational education, or vocational college/university) and family history of premature MI (yes, no). Other potential confounders such as diastolic blood pressure (mmHg) and the consumption (g/day) of fruits, vegetables, fish, fiber, saturated and unsaturated fatty acids were not included in the models, because they did not change the risk estimates with more than 10% after adjustment. Furthermore, separate models were performed with and without adjustments for predefined intermediate variables, i.e. total and HDL cholesterol (mmol/L), systolic blood pressure (mmHg), diabetes mellitus (yes/no), and body mass index (kg/m^2^) depending on the determinant under investigation.

Tests for heterogeneity were performed to evaluate differences between the two coronary diseases (AMI versus UAP) using the competing risks procedure in Stata. However, the standard error for the difference of the log-RRs from this procedure assumes independence of both estimated RRs which would overestimate the standard error and thus overestimate the *p *values for their difference. Therefore, these *p *values and the associated confidence intervals were estimated based on a bootstrapping method. The log-RRs were obtained from the bootstrap samples using Stata's competing risks procedure and recalculated for each bootstrap-replication. The confidence interval and *p *value of the differences in RR between AMI and UAP were then calculated from the replicated statistics using the accelerated bias corrected method in Stata. Each bootstrap analysis was based on 1,000 replications.

Participants with CHD at baseline (n = 347) and those who were lost to follow-up (n = 12) or migrated out of the Maastricht region before baseline (n = 26) were excluded from the analyses. In addition, participants with missing or inconsistent data on the main variables (n = 1,286) or confounders (n = 381) were excluded, leaving 19,096 participants for statistical analyses.

## Results

During a mean follow-up of 11.1 years, 420 participants developed incident AMI and 274 participants developed incident UAP. Compared with the total cohort, both types of cases had less favorable distributions of the lifestyle factors and other risk factors for coronary diseases (table 1). Besides slightly higher percentages of never drinkers among the cases, no differences were found in the median amount of alcoholic beverages consumed.

**Table 1:** Baseline Characteristics of the CAREMA Cohort in the Netherlands, 1987-1997

	Total cohort (n = 19,096)	AMI cases (n = 420)	UAP cases (n = 274)
**Demographics**			
	**mean (SD)**	**mean (SD)**	**mean (SD)**
Age at baseline (years)	40.9 (10.9)	48.8 (7.6)	50.1 (7.0)
			
**Sex**	**n (%)**	**n (%)**	**n (%)**
Men	8,956 (46.9)	328 (78.1)	190 (69.3)
Women	10,140 (53.1)	92 (21.9)	84 (30.7)
			
**Baseline cohort**			
PPHVZ (1987-1992)	13,674 (71.6)	366 (87.1)	237 (86.5)
MORGEN (1993-1997)	5,422 (28.4)	54 (12.9)	37 (13.5)
			

**Lifestyle factors**			
			
**Smoking**			
Never	6,598 (34.6)	57 (13.6)	55 (20.1)
Ex	4,960 (26.0)	93 (22.1)	86 (31.4)
Current	7,538 (39.5)	270 (64.3)	133 (48.5)
			
	**mean (SD)**	**mean (SD)**	**mean (SD)**
Number of cigarettes/day among current smokers	15.7 (8.8)	18.8 (8.0)	17.4 (7.9)
Number of years of smoking among current smokers	22.7 (10.5)	30.4 (8.8)	30.7 (9.1)
			
**Alcohol consumption**	**n (%)**	**n (%)**	**n (%)**
Never	2,391 (12.5)	63 (15.0)	40 (14.6)
Ex-drinker	257 (1.3)	13 (3.1)	7 (2.6)
Occasionally (<1 glass/week)	4,468 (23.4)	77 (18.3)	57 (20.8)
Regular (≥ 1 glasses/week)	11,980 (62.7)	267 (63.6)	170 (62.0)
			
	**median (5**^**th **^**- 95**^**th **^**perc)**	**median (5**^**th **^**- 95**^**th **^**perc)**	**median (5**^**th **^**- 95**^**th **^**perc)**
Number of glasses/day among regular drinkers	1.4 (0.3 - 5.0)	1.4 (0.3 - 5.7)	1.4 (0.3 - 6.1)
			
**Occupational activity**^**a**^	**n (%)**	**n (%)**	**n (%)**
None to light	13,849 (72.5)	304 (72.4)	207 (75.5)
Moderate to heavy	3,327 (17.4)	86 (20.5)	48 (17.5)
			
**Non-occupational activity**^**a**^			
None to light	8,264 (43.3)	171 (40.7)	115 (42.0)
Moderate to heavy	10,828 (56.7)	249 (59.3)	159 (58.0)
			
**Family history of premature MI**			
No	15,980 (83.7)	331 (78.8)	216 (78.8)
Yes	3,116 (16.3)	89 (21.2)	58 (21.2)
			

**Confounding/intermediate factors**			
			
**Level of education**			
Primary school/junior vocational education	11,251 (58.9)	316 (75.2)	208 (75.9)
Secondary vocational education	4,398 (23.0)	64 (15.2)	40 (14.6)
Vocational college/university	3,447 (18.1)	40 (9.5)	26 (9.5)
			
Diabetes mellitus	164 (0.9)	20 (4.8)	7 (2.6)
			
	**mean (SD)**	**mean (SD)**	**mean (SD)**
Systolic blood pressure (mmHg)	119.2 (14.3)	128.1 (16.6)	130.3 (17.1)
Total cholesterol (mmol/L)	5.4 (1.1)	6.3 (1.2)	6.3 (1.1)
HDL cholesterol (mmol/L)	1.3 (0.3)	1.0 (0.2)	1.1 (0.3)
Body mass index (kg/m^2^)	24.7 (3.9)	26.7 (4.0)	26.4 (3.9)
			

AMI, acute myocardial infarction; MI, myocardial infarction; MORGEN, Monitoring Project on Chronic Disease Risk Factors; n, number; perc, percentile; PPHVZ, Monitoring Project on Cardiovascular Risk Factors; SD, standard deviation; UAP, unstable angina pectoris.

^a ^Numbers may not add up to the total number of subjects because of missing values.

Compared with AMI cases, UAP cases were more often female (table 1) and slightly older at the time of diagnosis (table 2). Furthermore, table 2 shows that UAP cases more often suffered from two- or three-vessel disease although this observation might be biased by the higher percentage of AMI cases with unknown catheterization results.

**Table 2:** Disease Characteristics of the AMI and UAP Cases in the CAREMA Cohort, 1987-2003

	AMI cases (n = 420)	UAP cases (n = 274)
	**Mean (SD)**	**mean (SD)**
Age at diagnosis (years)	56.5 (7.7)	58.4 (7.4)
Time at risk (years)	7.2 (4.2)	7.9 (3.9)
		
	**n (%)**	**n (%)**
**Coronary angiography**		
No abnormalities	5 (1.2)	2 (0.7)
1-Vessel disease	107 (25.5)	84 (30.7)
2-Vessel disease	77 (18.3)	55 (20.1)
3-Vessel disease	61 (14.5)	62 (22.6)
Left main stem stenosis	9 (2.1)	16 (5.8)
Unknown^a^	161 (38.3)	55 (20.1)
		
**Follow-up**		
Revascularization within 60 days after diagnosis^b^	174 (41.4)	137 (50.0)
		
	**median (5**^**th **^**- 95**^**th **^**perc)**	**median (5**^**th **^**- 95**^**th **^**perc)**
Time to death (years)	2.9 (0.0 - 14.5)^c^	2.4 (0.0 - 14.2)
		

AMI, acute myocardial infarction; n, number; perc, percentile; SD, standard deviation; UAP, unstable angina pectoris.

^a ^No coronary angiography performed or findings not reported.

^b ^Exclusion of cases with a diagnosis of UAP (for the AMI cases) or AMI (for the UAP cases) within these 60 days and preceding the revascularization.

^c ^Restricted to cases with incident AMI who were not identified as case by the causes of death registry of Statistics Netherlands (n = 66).

### Lifestyle factors

Increased risks of both UAP and AMI were found in current and ex-smokers (table 3). A clear dose-response trend with smoking frequency was observed (*p *trend < 0.001 for AMI; 0.01 for UAP). The risks also increased with an increasing number of years smoked, although the dose-response trends were less obvious and only statistically significant for AMI. For all associations, RRs were higher for AMI than for UAP. All risks associated with smoking, except for the smoking duration, attenuated after adjustment for possible intermediate variables, but almost all remained highly significant.

**Table 3:** Multivariable Adjusted Rate Ratios for Smoking in the CAREMA Cohort, 1987-2003

	Acute Myocardial Infarction					Unstable Angina Pectoris					
			
	Cases/person-years	**Adjust. 1**^**a**^		**Adjust. 2**^**b**^		Cases/person-years	**Adjust. 1**^**a**^		**Adjust. 2**^**b**^		
					
		RR	95% CI	RR	95% CI		RR	95% CI	RR	95% CI	***P *heterogeneity**^**c**^
**Smoking status**											
Never	57/70,666	1	reference	1	reference	55/70,666	1	reference	1	reference	
Ex	93/54,638	1.30	0.93, 1.81	1.31	0.93, 1.83	86/54,638	1.33	0.94, 1.88	1.36	0.96, 1.93	
Current	270/84,269	3.38	2.53, 4.51	3.08	2.29, 4.14	133/84,269	1.77	1.28, 2.43	1.64	1.18, 2.27	<0.001
*P *trend			<0.001		<0.001			<0.001		0.003	
											
**Frequency (cig/day)**^d,e^											
Never	57/70,666	1	reference	1	reference	55/70,666	1	reference	1	reference	
>0 - 5	15/10,793	1.80	0.86, 3.79	1.88	0.89, 3.97	8/10,793	1.74	0.66, 4.55	1.89	0.71, 4.99	
>5 - 10	42/17,687	2.73	1.41, 5.31	2.59	1.33, 5.04	24/17,687	2.98	1.27, 7.01	2.95	1.24, 7.01	
>10 - 15	49/19,261	2.74	1.40, 5.35	2.54	1.30, 4.99	36/19,261	3.99	1.71, 9.34	3.91	1.65, 9.24	
>15 - 20	78/19,954	3.94	2.04, 7.61	3.20	1.65, 6.20	34/19,954	3.46	1.45, 8.27	2.94	1.22, 7.11	
>20	86/16,574	4.97	2.56, 9.64	3.98	2.05, 7.75	31/16,574	3.52	1.46, 8.49	3.06	1.25, 7.48	0.01
*P *trend			<0.001		<0.001			0.01		0.09	
											
Continuous, 5-cigarettes/day increments^f^	270/84,269	1.14	1.06, 1.21	1.08	1.01, 1.15	133/84,269	1.05	0.96, 1.16	1.00	0.90, 1.10	0.09
											
**Duration (yrs)**^e,g^											
Never	57/70,666	1	reference	1	reference	55/70,666	1	reference	1	reference	
>0 - 10	7/11,311	1.49	0.65, 3.45	1.73	0.74, 4.02	5/11,311	2.16	0.82, 5.67	2.56	0.97, 6.80	
>10 - 20	34/25,705	1.96	1.19, 3.23	2.10	1.27, 3.46	10/25,705	1.07	0.50, 2.29	1.14	0.54, 2.45	
>20 - 30	89/27,307	2.55	1.69, 3.85	2.73	1.80, 4.14	47/27,307	1.90	1.14, 3.18	2.03	1.21, 3.42	
>30 - 40	112/16,644	2.40	1.57, 3.65	2.51	1.63, 3.85	57/16,644	1.48	0.88, 2.48	1.52	0.89, 2.57	
>40	28/3,302	1.57	0.90, 2.71	1.93	1.10, 3.36	14/3,302	0.93	0.46, 1.89	1.05	0.51, 2.15	0.03
*P *trend			<0.001		<0.001			0.29		0.21	
											
Continuous, 10-years increments^f^	270/84,269	1.13	0.90, 1.42	1.13	0.90, 1.42	133/84,269	0.96	0.72, 1.28	0.94	0.71, 1.26	0.80
											
**Time since quitting (yrs)**^h^											
Never	57/70,666	1	reference	1	reference	55/70,666	1	reference	1	reference	
>5	61/36,603	0.87	0.55, 1.39	1.01	0.63, 1.62	53/36,603	0.92	0.54, 1.56	1.06	0.62, 1.80	
>2 - 5	15/7,476	1.44	0.70, 2.96	1.43	0.69, 2.95	14/7,476	1.76	0.78, 3.98	1.73	0.76, 3.95	
>0 - 2	15/9,392	1.34	0.64, 2.84	1.51	0.72, 3.18	17/9,392	1.97	0.87, 4.47	2.29	1.02, 5.16	
Current	265/82,188	2.75	1.58, 4.78	2.82	1.61, 4.91	130/82,188	1.66	0.84, 3.27	1.72	0.87, 3.41	0.003
*P *trend			<0.001		<0.001			0.03		0.06	

Adjust, adjustment; CI, confidence interval; cig, cigarettes; RR, rate ratio; yrs, years.

Table summary: Smoking increased the risk of both AMI and UAP. Dose-response relationships were seen with the number of cigarettes smoked per day and the number of smoking years although this trend was less obvious for the latter. All RRs were significantly higher for AMI than for UAP (*p *heterogeneity < 0.03). Furthermore, the risks of AMI and UAP decreased the longer ago subjects quitted smoking.

^a ^Rate ratios adjusted for age at baseline (years), sex, baseline cohort (PPHVZ or MORGEN), total alcohol consumption (glasses/day), diabetes mellitus (yes/no), level of education (primary school/junior vocational education, secondary vocational education or vocational college/university), and family history of premature MI (yes/no).

^b ^Additionally adjusted for intermediates: total and HDL cholesterol levels (mmol/L), systolic blood pressure (mmHg), and body mass index (kg/m^2^).

^c ^Heterogeneity test for the difference in the RRs between AMI and UAP. The *p *values were similar for the models with and without adjustments for intermediates.

^d ^Additionally adjusted for duration (years).

^e ^Exclusion of ex-smokers from the analyses.

^f ^Analyses restricted to current smokers.

^g ^Additionally adjusted for frequency (cigarettes/day).

^h ^Additionally adjusted for duration (years) and number of cigarettes/day smoked in the past.

The risk of AMI and UAP decreased the longer ago subjects quitted smoking (table 3). Ex-smokers who quitted more than five years ago had about the same risk as never smokers. For ex-smokers who stopped less than two years ago, the risk of AMI was lower than for current smokers, whereas the risk of UAP did not differ considerably for current smokers and ex-smokers who quitted less than five years ago.

Regular alcohol consumption decreased the risk of AMI and UAP, although this association with AMI was stronger than with UAP (table 4). While the risk of AMI decreased with the number of glasses consumed per day, the risk of UAP did not decrease further when more than four glasses were consumed per day. The protective effect of alcohol persisted after adjustment for intermediate variables. Furthermore, the effect was found for all types of alcoholic beverages consumed (table 4).

**Table 4:** Multivariable Adjusted Rate Ratios for Alcohol Consumption in the CAREMA Cohort, 1987-2003

	Acute Myocardial Infarction					Unstable Angina Pectoris					
			
	Cases/person-years	**Adjust. 1**^**a**^		**Adjust. 2**^**b**^		Cases/person-years	**Adjust. 1**^**a**^		**Adjust. 2**^**b**^		
					
		RR	95% CI	RR	95% CI		RR	95% CI	RR	95% CI	***P *heterogeneity**^**c**^
**Alcohol consumption**											
Never	63/26,819	1	reference	1	reference	40/26,819	1	reference	1	reference	
Ex	13/2,677	0.96	0.53, 1.77	0.99	0.54, 1.82	7/2,677	1.06	0.47, 2.40	1.10	0.49, 2.48	
Occasionally	77/48,218	0.86	0.61, 1.20	0.89	0.63, 1.25	57/48,218	1.04	0.69, 1.57	1.08	0.71, 1.62	
Regular	267/131,859	0.54	0.41, 0.73	0.65	0.48, 0.87	170/131,859	0.66	0.45, 0.95	0.72	0.49, 1.05	0.86
*P *trend			<0.001		0.002			0.008		0.05	
											
**Total alcohol consumption**^d ^**(glasses/day)**											
Never	63/26,819	1	reference	1	reference	40/26,819	1	reference	1	reference	
>0 - 2	165/90,441	0.60	0.44, 0.82	0.69	0.50, 0.94	107/90,441	0.71	0.48, 1.05	0.77	0.52, 1.15	
>2 - 4	69/29,221	0.48	0.33, 0.70	0.61	0.42, 0.90	39/29,221	0.55	0.34, 0.90	0.64	0.38, 1.05	
>4	33/12,197	0.40	0.26, 0.63	0.54	0.34, 0.86	24/12,197	0.70	0.40, 1.21	0.82	0.46, 1.46	0.89
*P *trend			<0.001		0.003			0.04		0.18	
											
Continuous, 1-glass/day increments^e^	267/131,859	0.91	0.85, 0.98	0.94	0.88, 1.01	170/131,859	0.98	0.90, 1.07	0.99	0.91, 1.08	0.70
											
**Beer consumption**^d,f ^**(glasses/day)**											
Never	63/26,819	1	reference	1	reference	40/26,819	1	reference	1	reference	
No beer	46/31,949	0.75	0.49, 1.15	0.85	0.55, 1.30	34/31,949	0.92	0.55, 1.53	1.00	0.60, 1.66	
>0 - 2	144/71,186	0.58	0.42, 0.81	0.65	0.47, 0.91	86/71,186	0.68	0.44, 1.03	0.73	0.47, 1.12	
>2 - 4	51/20,360	0.49	0.33, 0.73	0.62	0.41, 0.93	33/20,360	0.69	0.41, 1.16	0.79	0.46, 1.34	
>4	26/8,364	0.45	0.27, 0.73	0.58	0.35, 0.96	17/8,364	0.74	0.40, 1.39	0.84	0.44, 1.60	0.96
*P *trend			<0.001		0.005			0.12		0.28	
											
Continuous, 1-glass/day increments^e^	267/131,859	0.92	0.85, 0.99	0.94	0.87, 1.02	170/131,859	1.00	0.92, 1.09	1.01	0.93, 1.10	0.62
											
**Wine consumption**^d,f ^**(glasses/day)**											
Never	63/26,819	1	reference	1	reference	40/26,819	1	reference	1	reference	
No wine	177/63,871	0.67	0.47, 0.94	0.74	0.52, 1.04	103/63,871	0.73	0.47, 1.12	0.78	0.50, 1.22	
>0 - 2	85/63,013	0.60	0.42, 0.86	0.69	0.48, 1.00	60/63,013	0.68	0.44, 1.06	0.77	0.49, 1.20	
>2	5/4,975	0.34	0.13, 0.86	0.44	0.17, 1.12	7/4,975	0.79	0.34, 1.83	0.97	0.42, 2.26	0.82
*P *trend			0.002		0.02			0.11		0.30	
											
Continuous, 1-glass/day increments^e^	267/131,859	0.91	0.74, 1.11	0.95	0.77, 1.16	170/131,859	0.94	0.73, 1.19	0.97	0.77, 1.23	0.47
											
**Liquor consumption**^d,f ^**(glasses/day)**											
Never	63/26,819	1	reference	1	reference	40/26,819	1	reference	1	reference	
No liquor	200/100,509	0.70	0.50, 0.98	0.78	0.56, 1.09	126/100,509	0.73	0.48, 1.11	0.79	0.52, 1.21	
>0 - 2	60/29,754	0.50	0.34, 0.74	0.56	0.38, 0.84	42/29,754	0.59	0.36, 0.97	0.65	0.40, 1.06	
>2	7/1,596	0.46	0.20, 1.02	0.58	0.26, 1.30	2/1,596	0.27	0.06, 1.16	0.31	0.07, 1.30	0.97
*P *trend			<0.001		0.007			0.02		0.05	
											
Continuous, 1-glass/day increments^e^	267/131,859	0.89	0.71, 1.11	0.93	0.74, 1.17	170/131,859	0.68	0.47, 0.99	0.69	0.48, 1.01	0.23

Adjust, adjustment; CI, confidence interval; RR, rate ratio.

Table summary: Alcohol consumption was associated with a protective effect on the risk of both AMI and UAP, which was independent of the type of alcoholic beverage consumed. These associations seemed to be stronger for the risk of AMI than of UAP although not statistically significant (*p *heterogeneity > 0.05).

^a ^Rate ratios adjusted for age at baseline (years), sex, baseline cohort (PPHVZ or MORGEN), smoking status (never, ex- or current smoker), smoking frequency (cigarettes/day), smoking duration (years), total cholesterol level (mmol/L), diabetes mellitus (yes/no), level of education (primary school/junior vocational education, secondary vocational education or vocational college/university), family history of premature MI (yes/no), and body mass index (kg/m^2^).

^b ^Additionally adjusted for intermediates: HDL cholesterol level (mmol/L) and systolic blood pressure (mmHg).

^c ^Heterogeneity test for the difference in the RRs between AMI and UAP. The *p *values were similar for the models with and without adjustments for intermediates.

^d ^Exclusion of ex- and occasional drinkers from the analyses.

^e ^Analyses restricted to regular drinkers (≥1 glass/week).

^f ^Additionally adjusted for the other alcoholic beverages.

For both occupational and non-occupational physical activity in relation to CHD, sex was found to be a significant effect modifier. In men, occupational activity seemed to be a risk factor for both endpoints (table 5). In women, however, moderate to heavy activity was associated with a lower risk of AMI and UAP, although these estimates were based on small numbers of cases. Non-occupational activity seemed to be protective in men, which attenuated after adjustment for intermediate variables. In women, however, non-occupational activity was found to be a risk factor. This association became stronger after adjustment for intermediate variables, but was only statistically significant for AMI.

**Table 5:** Multivariable Adjusted Rate Ratios for Physical Activity in the CAREMA Cohort, 1987-2003

	Acute Myocardial Infarction					Unstable Angina Pectoris					
			
	Cases/person-years	**Adjust. 1**^**a**^		**Adjust. 2**^**b**^		Cases/person-years	**Adjust. 1**^**a**^		**Adjust. 2**^**b**^		
					
		RR	95% CI	RR	95% CI		RR	95% CI	RR	95% CI	***P *heterogeneity**^**c**^
**Occupationally**^d^											
***Men***											
None to light activity	222/65,190	1	reference	1	reference	132/65,190	1	reference	1	reference	
Moderate to heavy activity	80/22,849	1.27	0.97, 1.66	1.37	1.05, 1.80	41/22,849	1.15	0.79, 1.66	1.21	0.84, 1.76	0.49
***Women***											
None to light activity	82/88,459	1	reference	1	reference	75/88,459	1	reference	1	reference	
Moderate to heavy activity	6/16,078	0.48	0.21, 1.10	0.57	0.25, 1.32	7/16,078	0.67	0.31, 1.48	0.72	0.33, 1.59	0.89
											
**Non-occupationally**^e^											
***Men***											
None to light activity	115/30,536	1	reference	1	reference	72/30,536	1	reference	1	reference	
Moderate to heavy activity	187/57,503	0.91	0.71, 1.15	1.03	0.81, 1.31	101/57,503	0.69	0.51, 0.95	0.74	0.54, 1.02	0.41
***Women***											
None to light activity	32/45,070	1	reference	1	reference	28/45,070	1	reference	1	reference	
Moderate to heavy activity	56/59,466	1.57	1.00, 2.46	1.81	1.15, 2.87	54/59,466	1.42	0.88, 2.27	1.55	0.96, 2.49	0.76

Adjust, adjustment; CI, confidence interval; RR, rate ratio.

Table summary: Occupational physical activity seemed to be a risk factor for both AMI and UAP in men, while in women it was associated with lower risks of both coronary endpoints. However, these associations were only statistically significant in men for the risk of AMI after adjustment for possible intermediates. Non-occupational physical activity seemed to be protective in men, while it was found to be a risk factor in women, although these associations were only statistically significant for the risk of AMI in women. Associations seemed to be stronger with the risk of AMI than of UAP, although not statistically significant (*p *heterogeneity > 0.05).

^a ^Rate ratios adjusted for age at baseline (years), baseline cohort (PPHVZ or MORGEN), smoking status (never, ex- or current smoker), smoking frequency (cigarettes/day), smoking duration (years), total alcohol consumption (glasses/day), level of education (primary school/junior vocational education, secondary vocational education or vocational college/university), and family history of premature MI (yes/no).

^b ^Additionally adjusted for intermediates: total and HDL cholesterol levels (mmol/L), diabetes mellitus (yes/no), systolic blood pressure (mmHg), and body mass index (kg/m^2^).

^c ^Heterogeneity test for the difference in the RRs between AMI and UAP. The *p *values were similar for the models with and without adjustments for intermediates.

^d ^Additionally adjusted for non-occupational physical activity.

^e ^Additionally adjusted for occupational physical activity.

### Family history

Compared with subjects with no parents affected, the highest risks of both coronary endpoints were found for subjects with both parents affected, especially when they were diagnosed at younger age (table 6). Both these trends were stronger for UAP than for AMI. Clear dose-response trends in risk were found for the combination of the number of parents affected with their age at diagnosis, except for the last category and the risk of AMI which may be due to the very low number of cases in this category (table 6).

**Table 6:** Multivariable Adjusted Rate Ratios for Family History in the CAREMA Cohort, 1987-2003

	Acute Myocardial Infarction			Unstable Angina Pectoris			
			
	Cases/person-years	RR	95% CI	Cases/person-years	RR	95% CI	***P *heterogeneity**^**a**^
**Number of parents affected**							
No parents affected	253/147,248	1	reference	148/147,248	1	reference	
One parent affected	144/56,391	1.34	1.09, 1.65	106/56,391	1.70	1.32, 2.18	
Both parents affected	25/6,000	1.79	1.18, 2.71	19/6,000	2.22	1.37, 3.59	0.33
*P *trend			<0.001			<0.001	
							
**Age at diagnosis of parents**							
No parents affected	253/147,248	1	reference	148/147,248	1	reference	
Non-premature MI	80/29,244	1.16	0.90, 1.50	68/29,244	1.62	1.21, 2.16	
Premature MI^b^	89/33,146	1.70	1.33, 2.17	57/33,146	1.97	1.44, 2.69	0.16
*P *trend			<0.001			<0.001	
							
**Number of parents affected including age at diagnosis**							
No parents affected	253/147,248	1	reference	148/147,248	1	reference	
One parent affected, non-premature MI	74/27,105	1.18	0.91, 1.53	61/27,105	1.60	1.18, 2.16	
Both parents affected, both non-premature MI	6/2,139	1.03	0.46, 2.33	7/2,139	1.79	0.83, 3.83	
One parent affected, premature MI	70/29,285	1.58	1.21, 2.06	45/29,285	1.85	1.32, 2.60	
Both parents affected, one premature MI	17/2,279	3.04	1.85, 4.99	8/2,279	2.42	1.19, 4.96	
Both parents affected, both premature MI	2/1,582	0.78	0.19, 3.13	4/1,582	2.94	1.08, 7.99	0.51
*P *trend			<0.001			<0.001	

CI, confidence interval; MI, myocardial infarction; RR, rate ratio. Rate ratios adjusted for age at baseline (years), sex, baseline cohort (PPHVZ or MORGEN), smoking status (never, ex- or current smoker), smoking frequency (cigarettes/day), smoking duration (years), total alcohol consumption (glasses/day), total and HDL cholesterol levels (mmol/L), diabetes mellitus (yes/no), systolic blood pressure (mmHg), and body mass index (kg/m^2^).

Table summary: A positive family history is associated with increased risks of both AMI and UAP, especially when both parents are affected or when they are diagnosed at a younger age (premature MI). These associations seemed to be stronger for the risk of UAP than of AMI but the differences were not statistically significant (*p *heterogeneity > 0.05).

^a ^Heterogeneity test for the difference in the RRs between AMI and UAP.

^b ^Defined as age at diagnosis of MI ≤60 years for the father and ≤65 years for the mother. If both parents were affected, at least one parent had a premature MI.

On a multiplicative scale, none of the interactions between family history of premature MI and the lifestyle factors were statistically significant for both coronary diseases, except for occupational physical activity and the AMI risk in women. Compared with never smokers without a family history, the highest risks of AMI and UAP were found in current smokers with a positive family history (table 7). For AMI, the risk for current smokers without a family history was higher compared with ex-smokers with a family history, while the risk of UAP was higher for ex-smokers with a family history. For alcohol consumption, the largest protective effect was found for regular drinkers without a family history. In subjects with a positive family history, the protective effect of alcohol consumption is stronger for AMI than UAP.

**Table 7:** Multivariable Adjusted Rate Ratios for Smoking, Alcohol Consumption, and Physical Activity by Family History of Premature MI in the CAREMA Cohort, 1987-2003

	Acute Myocardial Infarction						Unstable Angina Pectoris					
		
	No family history (n = 15,980)			**Family history**^**a **^**(n = 3,116)**			No family history (n = 15,980)			Family history (n = 3,116)		
				
	Cases/person-years	RR	95% CI	Cases/person-years	RR	95% CI	Cases/person-years	RR	95% CI	Cases/person-years	RR	95% CI
												
**Smoking status**^b^												
Never	43/60,695	1	reference	14/9,971	1.78	0.96, 3.28	43/60,695	1	reference	12/9,971	1.71	0.89, 3.29
Ex	76/45,928	1.36	0.93, 1.98	17/8,710	2.01	1.14, 3.55	68/45,928	1.34	0.90, 1.98	18/8,710	2.50	1.43, 4.37
Current	212/69,412	3.11	2.22, 4.36	58/14,857	5.25	3.51, 7.84	105/69,412	1.63	1.13, 2.36	28/14,857	2.86	1.76, 4.64
*P *interaction^c^						0.89						0.97
												
**Alcohol consumption**^d^												
Never	49/22,647	1	reference	14/4,173	1.71	0.94, 3.11	33/22,647	1	reference	7/4,173	1.44	0.63, 3.25
Ex	9/2,229	0.86	0.42, 1.77	4/448	2.58	0.92, 7.20	6/2,229	1.10	0.46, 2.66	1/448	1.49	0.20, 10.98
Occasionally	60/40,094	0.91	0.62, 1.33	17/8,123	1.42	0.81, 2.48	42/40,094	0.98	0.62, 1.56	15/8,123	2.16	1.16, 4.01
Regular	213/111,065	0.66	0.47, 0.92	54/20,794	1.05	0.70, 1.58	135/111,065	0.70	0.46, 1.05	35/20,794	1.21	0.73, 2.00
*P *interaction						0.79						0.84
												
**Occupational activity**^e^												
***Men***												
None to light	185/55,603	1	reference	37/9,587	1.47	1.03, 2.11	111/55,603	1	reference	21/9,587	1.49	0.93, 2.40
Moderate to heavy	56/18,971	1.23	0.90, 1.68	24/3,878	2.92	1.88, 4.54	30/18,971	1.13	0.74, 1.72	11/3,878	2.35	1.24, 4.45
*P *interaction						0.12						0.44
***Women***												
None to light	67/73,701	1	reference	15/14,758	1.08	0.61, 1.92	57/73,701	1	reference	18/14,758	1.79	1.04, 3.09
Moderate to heavy	2/13,319	0.24	0.06, 0.99	4/2,759	1.98	0.71, 5.55	5/13,319	0.72	0.29, 1.83	2/2,759	1.29	0.31, 5.36
*P *interaction						0.03						0.99
												
**Non-occupational activity**^e^												
***Men***												
None to light	91/25,465	1	reference	24/5,071	1.48	0.94, 2.34	58/25,465	1	reference	14/5,071	1.53	0.85, 2.76
Moderate to heavy	150/49,109	0.97	0.75, 1.27	37/8,394	1.86	1.26, 2.74	83/49,109	0.72	0.51, 1.03	18/8,394	1.27	0.74, 2.17
*P *interaction						0.40						0.73
***Women***												
None to light	28/36,894	1	reference	4/8,176	0.58	0.20, 1.70	20/36,894	1	reference	8/8,176	1.86	0.81, 4.30
Moderate to heavy	41/50,126	1.43	0.87, 2.38	15/9,340	2.75	1.45, 5.23	42/50,126	1.57	0.91, 2.72	12/9,340	2.75	1.33, 5.67
*P *interaction						0.06						0.91
												

CI, confidence interval; n, number; RR, rate ratio. All RRs were adjusted for age at baseline (years), baseline cohort (PPHVZ or MORGEN), level of education (primary school/junior vocational education, secondary vocational education or vocational college/university), diabetes mellitus (yes/no), total and HDL cholesterol levels (mmol/L), systolic blood pressure (mmHg), and body mass index (kg/m^2^).

Table summary: None of the interactions between family history of premature MI and the lifestyle factors smoking, alcohol consumption, and physical activity were statistically significant on a multiplicative scale for both AMI and UAP, except for occupational physical activity and the AMI risk in women (*p *interaction 0.03). Nevertheless, the highest risks of both coronary endpoints were found in subjects with both a positive family history and the most unfavorable level of the lifestyle factors.

^a ^Positive family history was defined as at least one parent with a premature MI, i.e. age at diagnosis ≤60 years for the father and ≤65 years for the mother.

^b ^RRs additionally adjusted for sex and alcohol consumption (glasses/day).

^c ^*P *interaction refers to the significance of the interaction between the determinant and family history of premature MI on a multiplicative scale.

^d ^RRs additionally adjusted for sex, smoking status (never, ex- or current smoker), smoking frequency (cigarettes/day), and smoking duration (years).

^e ^RRs additionally adjusted for smoking status (never, ex- or current smoker), smoking frequency (cigarettes/day), smoking duration (years), and alcohol consumption (glasses/day). Furthermore, occupational and non-occupational physical activity were mutually adjusted.

For occupational physical activity, the highest risks of AMI and UAP were found in subjects with a positive family history and a moderate to heavy activity level, although these risks were only statistically significant in men (table 7). In women, the effect of occupational activity on the risk of AMI differed between subjects with and without a family history (*p *interaction 0.03), although this interaction was based on a very low number of cases in the moderate to heavy activity category. In men, no differences in the effect of non-occupational activity were seen between subjects with and without a family history for both coronary diseases. In women, however, there seemed to be a synergistic effect between non-occupational activity and family history for the risk of AMI with the highest risk for women with a positive family history and a moderate to heavy physical activity level, although this interaction was not statistically significant.

## Discussion

In this prospective study, the effects of smoking, alcohol consumption, and physical activity pointed in the same direction for AMI and UAP. The effects, however, were nearly always stronger for AMI than UAP, although these differences in risks between the two coronary diseases were only statistically significant for the associations with smoking. For both coronary diseases, positive trends in risk were found with the number of cigarettes smoked. These trends were also observed for the number of years smoked, although less obvious. For alcohol consumption, a decreasing trend in risk was found for both coronary diseases. This trend was independent of the type of alcoholic beverage consumed. For occupational and non-occupational physical activity, opposite effects on the risks of AMI and UAP were found between men and women.

Clear positive trends in risk were found for both the number of parents affected with an MI and for the age at diagnosis of the parents. The highest risk was found for subjects with both parents affected of whom at least one parent had a premature MI. As opposed to the lifestyle factors, the associations with family history seemed to be stronger for UAP than AMI, although the differences in risks were not statistically significant. Furthermore, similar effects of the lifestyle factors were found for subjects with and without a family history of premature MI.

### Strengths and limitations

The response rate of this prospective cohort study was 43%, which is not uncommon for this kind of studies. Furthermore, a large variation in the exposure status is more important to investigate etiological associations than having a representative study population. In prospective cohort studies, the completeness of follow-up is essential. In this study, only twelve participants (0.1%) were lost to follow-up. Therefore, exposure-related loss to follow-up is unlikely. The cardiologic follow-up is also expected to be nearly complete because of the central and unique position of the UHM in the study region. Furthermore, the cardiologic data were collected in an extensive manner which is probably less susceptible to misclassification [34].

During follow-up, more sensitive screening tests became available for the diagnosis of AMI which may have decreased the interchange between a diagnosis of AMI and of UAP. This interchange would have attenuated the differences in effects between AMI and UAP. However, as mentioned above, the way in which the AMI and UAP cases were identified is probably more accurate than using data from other registries such as hospital discharge registries [34]. Furthermore, no change in the ratio between the number of cases diagnosed with AMI and with UAP was seen before and after the introduction of the screening tests.

According to the redefinition of AMI by the European Society of Cardiology and the American College of Cardiology [35], STEMI and non-STEMI can be considered as separate pathophysiological entities. This may suggest that the associations with the lifestyle factors may differ between STEMI and non-STEMI. As no sufficient data on ECG abnormalities was available at the time of the statistical analyses to distinct between STEMI and non-STEMI, separate analyses for these subtypes of AMI could not be performed within this study.

In this cohort study, family history and the lifestyle factors were self-reported by the participants, which may have led to exposure misclassification. However, several studies have shown that self-reported data can be used quite accurately to define family history [36,37] and smoking status [38]. In addition, Pols et al. have shown that the questions on physical activity in the MORGEN study were suitable for ranking the participants according to their physical activity level [39]. Because the exposure measurements took place before the occurrence of the disease, the misclassification is probably non-differential [40]. Therefore, the use of self-reported data has probably not biased our results to a great extent. However, two validation studies within subsamples of our study population found that the amount of alcohol consumption was underestimated by the food frequency questionnaire [41,42]. This misclassification may have resulted in an underestimation of the effect of alcohol consumption in our study.

The participants included in this study were relatively young at baseline (20-59 years). Consequently, both the AMI and UAP cases were diagnosed at a relatively young age. This may have affected the RRs. As the risks of these coronary diseases increase with an increasing age, the difference in absolute risks between the exposed and unexposed participants becomes relatively smaller at an older age, resulting in a lower RR. Therefore, the RRs in our study may be higher compared to other studies in which the study population and cases are relatively older.

### Comparison with previous studies

Many studies investigated the associations between smoking, alcohol consumption, and physical activity and the risk of coronary diseases, part of which are summarized in several reviews/meta-analyses, e.g. [9,12-18]. However, these studies did not discern between the risk of AMI and UAP and will, therefore, not be discussed any further.

To our knowledge, two studies investigated the effect of smoking status on UAP risk [2,3]. In a case-control study by Sagastagoitia et al. [2], smoking was found to be independently associated with the presence of UAP (OR 3.42, 95% CI 1.77-6.58). This OR is considerably higher than the RR of 1.64 found in the present prospective study which may be due to differences in study design. Kennon et al. [3] found that smokers were at higher risk to be discharged with a diagnosis of AMI than of UAP (OR 1.49, 95% CI 1.09-2.03) which suggests that the association with smoking is stronger for AMI than UAP as was found in the present study. No studies investigated the effects of smoking frequency or duration on the UAP risk.

Five studies found an inverse association between alcohol consumption and the risk of angina pectoris [4-8], although they did not specifically look at UAP. In the present study, the association seemed to be stronger for AMI than UAP which was also found in the study by Marques-Vidal et al. for angina pectoris [8], while the other studies found no great differences between the two coronary diseases. However, comparisons between the studies should be done with caution because of differences in disease definition and in average alcohol consumption.

The results of studies that investigated the association between occupational or non-occupational physical activity and the risk of angina pectoris are still inconclusive [9-11]. In a meta-analysis by Berlin et al. [9], occupational activity was found to be a significant protective factor for angina but only when the group with the highest activity was compared with the moderately active group (RR 0.6, 95% CI 0.4-0.9). Contrary to our expectations, occupational activity seemed to increase the UAP risk in our study, but only in men. Although not completely clear, this finding may be due to residual confounding, the limited contrast in the activity level between the comparison groups or chance. For non-occupational activity, no association was found with the risk of angina by Berlin et al. [9], while Wagner et al. [11] found a significant positive association (RR 1.07, 95% CI 1.02-1.12). However, these studies differed in the categorizing of physical activity into comparison groups which complicates their comparison. In the present study, the inclusion of subjects with a light activity level in the reference group may be the reason for not finding a protective effect of higher activity levels on the coronary disease risk.

To our knowledge, only three studies investigated the association between family history and the risk of angina [30,43,44] of which one study specifically looked at UAP [30]. In line with the present study, two studies [30,43] also found higher risks for angina than for AMI in subjects with a positive family history, while the other study [44] found no differences in risks. However, in all these studies, different definitions of family history were used which complicates their comparison.

In this study, only a statistically significant interaction on a multiplicative scale was found between occupational physical activity and family history of a premature MI for the risk of AMI in women. Five studies also investigated a possible synergistic effect between smoking and family history [27-30]. One study [29] also found no synergistic effect, while in two other studies this interaction was only significant in women although these studies assessed the interaction on an additive scale [27,28]. In addition, these two studies also found no synergistic effect between physical inactivity and family history [27,28] as was found in this study for non-occupational physical activity and occupational activity in men. Nevertheless, subjects with a positive family history and the most unfavorable level of lifestyle factors had the highest risks in all of these studies, including our study.

### Risk prediction

This study and other studies have shown that lifestyle factors such as smoking, physical inactivity, and alcohol consumption play an important role in the etiology of coronary diseases [1]. Therefore, changing the prevalences of these factors plays a role in the primary prevention of CHD. In current risk scores, however, smoking frequency and duration are mostly not included while some studies, including this study, found a clear dose-response relationship between the number of cigarettes smoked and the risk of cardiovascular disease e.g. [1,13]. In addition, alcohol consumption is mostly even neglected in these risk scores, whereas physical activity is only included in the risk score of the ARIC study [45]. Although family history of premature CHD or MI was found to be an independent risk factor for coronary diseases in several studies [25-27], this factor is only included in part of the current risk scores [46-51]. However, although these factors have been found to be strong independent risk factors for CHD, this does not mean that they have an added value to the risk prediction of CHD. Some studies which took these factors into consideration did not include them in the final algorithm because of limited availability [52] or limited predictive ability [47]. Still, focusing on these factors in both low- and high-risk subjects may help to prevent CHD.

## Conclusions

In this prospective cohort study, smoking, alcohol consumption, and physical activity affected the risk of both AMI and UAP. However, the strength of these associations seemed to differ between these two coronary diseases in which they were mostly stronger for AMI, although the differences in risks were only statistically significant for smoking. Opposed to this, the association with family history of MI seemed to be stronger for UAP. Nevertheless, more research is needed to elucidate these associations, especially for the risk of UAP. Although no synergistic effects on a multiplicative scale were found between the lifestyle factors and family history, the highest risks were found in subjects with both a positive family history and the most unfavorable level of the lifestyle factors. Therefore, future studies should evaluate whether changes in the prevalences of these lifestyle factors result in lower incidence rates of AMI and UAP and thus benefit the primary prevention of both coronary diseases, especially in subjects with a positive family history.

## Abbreviations

AMI: Acute Myocardial Infarction; CAG: Coronary Angiography; CAREMA: Cardiovascular Registry Maastricht; CHD: Coronary Heart Disease; CI: Confidence Interval; Cig: Cigarettes; CIS: Cardiology Information System; HDL: High-Density Lipoprotein; ICD: International Classification of Diseases; MI: Myocardial Infarction; MORGEN: Monitoring Project on Chronic Disease Risk factors; N: Number; OR: Odds Ratio; Perc: Percentile; PPHVZ: Monitoring Project on Cardiovascular Risk Factors; RR: Rate Ratio; SD: Standard Deviation; UHM: University Hospital Maastricht; UAP: Unstable Angina Pectoris; Yrs: Years.

## Competing interests

The authors declare that they have no competing interests.

## Authors' contributions

All authors of the research paper have participated in the writing of the manuscript, in the planning, execution or analysis of the study and have read and approved the final version submitted. The author's responsibilities were as follows: AHHM acquired the data, analyzed and interpreted data, performed statistical analyses, and drafted the manuscript. JMAB acquired the data, analyzed and interpreted data, was responsible for critical revision of the manuscript for important intellectual content, and supervised the study. LJS was responsible for the study concept and design, acquired the data, analyzed and interpreted data, was responsible for critical revision of the manuscript for important intellectual content, and supervised the study. EJMF was responsible for the study concept and design, acquired the data, was responsible for critical revision of the manuscript for important intellectual content, obtained funding, and supervised the study. WMMV acquired the data and was responsible for critical revision of the manuscript for important intellectual content. APMG was responsible for the study concept and design, acquired the data, was responsible for critical revision of the manuscript for important intellectual content, and supervised the study. PAVDB was responsible for the study concept and design, acquired the data, was responsible for critical revision of the manuscript for important intellectual content, obtained funding, and supervised the study.

## Pre-publication history

The pre-publication history for this paper can be accessed here:

http://www.biomedcentral.com/1471-2261/11/13/prepub
